# The mental health of all children in contact with social services: a population-wide record-linkage study in Northern Ireland

**DOI:** 10.1017/S2045796023000276

**Published:** 2023-05-16

**Authors:** Sarah McKenna, Dermot O’Reilly, Aideen Maguire

**Affiliations:** Administrative Data Research Centre Northern Ireland (ADRC-NI), Queen’s University Belfast, Belfast, Northern Ireland

**Keywords:** children’s social care, data linkage, mental health

## Abstract

**Aims:**

Children in contact with social services are at high risk for mental ill health, but it is not known what proportion of the child population has contact with social services or how risk varies within this group compared to unexposed peers. We aim to quantify the extent and nature of contact with social services within the child population in Northern Ireland (NI) and the association with mental ill health. We also examine which social care experiences identify those most at risk.

**Methods:**

This is a population-based record-linkage study of 497,269 children (aged under 18 years) alive and resident in NI in 2015 using routinely collected health and social care data. Exposure was categorized as (1) no contact, (2) referred but assessed as not in need (NIN), (3) child in need (CIN) and (4) child in care (CIC). Multilevel logistic regression analyses estimated odds ratios (ORs) for mental ill health indicated by receipt of psychotropic medication (antidepressants, anxiolytics, antipsychotics and hypnotics), psychiatric hospital admission and hospital-presenting self-harm or ideation.

**Results:**

Over one in six children (17.2%, *n* = 85,792) were currently or previously in contact with social services, and almost one child in every 20 (4.8%, *n* = 23,975) had contact in 2015. Likelihood of any mental ill health outcome increased incrementally with the level of contact with social services relative to unexposed peers: NIN (OR 5.90 [95% confidence interval (CI) 5.10–6.83]), CIN (OR 5.99 [95% CI 5.50–6.53]) and CIC (OR 12.60 [95% CI 10.63–14.95]). All tiers of contact, number of referrals, number of care episodes and placement type were strongly associated with the likelihood of mental ill health.

**Conclusion:**

Children who have contact with social services account for a large and disproportionate amount of mental ill health in the child population. Likelihood of poor mental health across indicators is highest in care experienced children but also extends to the much larger population of children in contact with social services but never in care. Findings suggest a need for targeted mental health screening and enhanced support for all children in contact with social services.

## Introduction

Children in contact with social services (i.e. subject to a referral, child in need [CIN] plan, child protection measures or in care) are a vulnerable population typically exposed to one or multiple adverse childhood experiences (Fitzsimons *et al.*, 2022; Turney *et al.*, 2017) that place them at increased risk of poor mental health (Gilbert *et al.*, 2009; Hughes *et al.*, 2017). The number of children in contact with social services in the UK is increasing, disproportionate to population growth (Fitzsimons *et al.*, 2022) and improving outcomes for children from adverse backgrounds is a UK policy priority (UK Government, 2021). Yet, there is a lack of population-wide evidence on the extent and nature of contact with children’s social care and on mental health outcomes based on different tiers of contact (Children’s Commissioner for England, 2017; House of Commons Education Committee, 2016).

Understanding the potential scale of the public health problem requires robust data on levels of exposure within the population and associated outcomes. Official statistical publications quantify the number of children in contact with social care at a specific census point each year and trends over time (Department for Education, 2022). Quantifying cumulative exposure at the population level is more challenging but essential given that children often enter, exit and re-enter social care as needs escalate and de-escalate. Statutory data in England have been used longitudinally in conjunction with population estimates to model the proportion of children assessed as in need of social care services. An estimated one in 10 children in 2018 needed a social worker in the previous 6 years (Department for Education, 2019) and an estimated one in seven children in 2019 over the previous 8 years (Fitzsimons *et al.*, 2022). However, these are estimates only. It is important to note that thresholds for intervention from statutory social services are generally high (MacAlister, 2021) and that a large proportion of referrals do not result in the provision of any service (Bunting *et al.*, 2018; UK Government, 2022). There is a paucity of studies based on population data, and it is not known what proportion of the child population has experienced a referral to social services or who has been assessed as not in need (NIN) of any help or support.

There is considerable debate as to whether care has a harmful, neutral or beneficial effect, as selection bias and confounding by indication are pervasive problems in studies of outcomes associated with care (Brännström *et al.*, 2020). It is not possible to establish causation from observational studies, but policy formation also requires evidence of need, which is the main aim of this study. Extant systematic reviews have consistently documented high levels of mental ill health among children in contact with social services compared with unexposed peers (Bronsard *et al.*, 2016; Engler *et al.*, 2022; Goemans *et al.*, 2016). However, most of the evidence base is from the USA, is based on relatively small samples, and has had a principal focus on children in care. A limited number of UK population-wide studies have found increased rates of self-harm, outpatient psychiatry visits, psychiatric hospitalizations and psychotropic prescribing among children in care compared to children never in care (Allik *et al.*, 2022; Fleming *et al.*, 2021). There have been fewer studies examining the mental health of children receiving support or protection at home or those with maltreatment allegations (Goemans *et al.*, 2016; Green *et al.*, 2020; Maclean *et al.*, 2019; O’Hare *et al.*, 2021). Studies of maltreated children or cases where maltreatment is suspected are important, but in the UK, these represent a minority of children who have contact with social services (Rodgers and McCluney, 2021).

Consequently, there is a need for population-level evidence on the extent and nature of contact with social services, including those below the tier of child protection and those referred but assessed as NIN of any help or support and the associated prevalence and risk of mental ill health. Furthermore, children in contact with social services are not a homogenous group, and their mental health status is likely to vary with aspects of their social care experiences, including the reason referred or placed in care, age at first referral or care entry, number of care episodes or placement type. However, evidence concerning the potential contribution of social care experiences to mental health is largely absent in existing studies or provides mixed results (Engler *et al.*, 2022; McKenna *et al.*, 2021; Xu and Bright, 2018). Northern Ireland (NI) has an integrated health and social care system allowing for easy linkage of population-wide social care data to a range of health-related datasets to better understand the mental health status of all children in contact with social services. The aims of this study are therefore the following:

1. To quantify the extent and nature of contact with social services in the child population in NI.
2. To determine the prevalence of mental ill health in all children in contact with social services, including those assessed as NIN of services and their general population peers.
3. To quantify the association between level of contact with social services and childhood mental ill health.
4. To examine which social care experiences may identify those most at risk (specifically reason for involvement or placement in care, age at first referral or placement in care, number of referrals or care episodes and placement type).

## Methods

### Population and data sources

This is a population-wide record-linkage study of all children, aged 17 years or less, who were alive and resident in NI on January 1, 2015. NI has a ‘free-at-the-point-of-contact’ health and social care system, which includes free prescription medications, and therefore almost the entire population are registered with a local General Practitioner (GP). GP patient data, including basic demographic information, is collated within the National Health Applications and Infrastructure Services (NHAIS), which provided the population spine. Reporting follows the Reporting of Studies Conducted using Observational Routinely collected Health Data (RECORD) guidelines (Benchimol *et al.*, 2015).

#### Social services data

The Social Services Client Administration and Retrieval Environment (SOSCARE) database provided a social care history for every individual from birth to December 31, 2015. Social services contact was classified into four mutually exclusive groups, based on highest level: (i) no contact, (ii) referred but assessed as not in need of help or protection, in every interaction with social services (NIN), (iii) assessed as a child in need (CIN) (i.e. subject to a CIN plan or child protection measures in their own home) and (iv) child in care (CIC) (i.e. foster, kinship, residential care or placed with parent). Although the legal definition of a CIC is a ‘looked after child’, consultation with service users identified a strong preference for the term ‘child in care’, which is therefore used throughout. Children in care are by definition also a CIN but are examined here as a separate group. To examine the mental health of children ‘currently’ in contact with social services and those with previous contact, the groups were subdivided into NIN, CIN and CIC prior to 2015 and during 2015.

SOSCARE data were also used to derive experiences related to social services contact: most frequent reason referred, most frequent reason in care, age first referred, age first placed in care, number of referral episodes, number of care episodes and most frequent care placement type (foster care with strangers, kinship foster care, children’s home and other). The most frequent reason each child had been referred to social services across their full social care history was established and grouped into three categories: parent or guardian factors (e.g. carer requires support); well-being prejudiced (e.g. subject to a child protection investigation) and other (e.g. awaiting assessment). The most frequent reason in care was also grouped into three categories: parent/guardian factors (e.g. physical or mental ill health, predicted inadequacy and incarceration), neglect or abuse (emotional, sexual, physical; suspected abuse) and other (e.g. relief of parental stress). Care episodes may relate to both new referrals after a case closure but also a change in the legal status of the child or a change in care provision such as a placement move.

#### Mental health data

Indicators of mental ill health were obtained through linkage to data for dispensed psychotropic medications from the Enhanced Prescribing Database, described in detail elsewhere (Maguire *et al.*, 2013), psychiatric hospital admission data and self-harm/ideation data from the NI Registry of Self-Harm, which records information on all presentations to hospital emergency departments (ED) for self-harm or self-harm/suicidal ideation (Public Health Agency, 2018). Mental ill health in 2015 was denoted by (i) receipt of at least one prescription for psychotropic medication (antidepressants, anxiolytics, hypnotics, antipsychotics and any psychotropic medication); (ii) an ED presentation for self-harm or self-harm/suicidal ideation (hereafter ideation); (iii) admission to a psychiatric hospital/ward and (iv) a summary ‘any mental ill health’ measure representing any of the first three categories. The four medication categories were non-exclusive. We excluded medications for treating substance misuse due to the rarity and complexity of these disorders, i.e. addiction can be both a symptom of and a cause of other mental health conditions. The indicator for ‘any psychotropic medication’ counted individuals only once. Due to the relative rarity of psychiatric admissions, it was not examined independently but only included in the summary ‘any mental ill health’ indicator.

#### Covariates

Other covariates for the child were derived from NHAIS and included age (defined at January 1, 2015, and included as a continuous variable in regression analyses), sex and area-level characteristics. Urban/rural residence was based on a classification of settlements in NI and grouped into urban (comprising the two largest cities), intermediate and rural (NISRA, 2015). A measure of disadvantage was extracted from the income deprivation domain of the NI Multiple Deprivation Measure, which provides information on the proportion of the population in each area living in households in receipt of income-related benefits and tax credits in 2010 (NISRA, 2010). It was divided into two groups (more deprived included the top two most deprived quintiles and less deprived contained all other areas). Health and social care in NI is divided into five Health and Social Care Trusts (HSCT), each managing the populations within their geographical area (Northern, Western, Southern, South-Eastern and Belfast). The HSCT each individual was resident in was determined from their address. Individual records were linked across all datasets using Health and Care Number, a unique identifier recorded in any interaction with health and social care services. Meta data describing the predictor and outcome variables and related coding is provided in Supplementary Table S1.

#### The cohort

**Figure 1.**
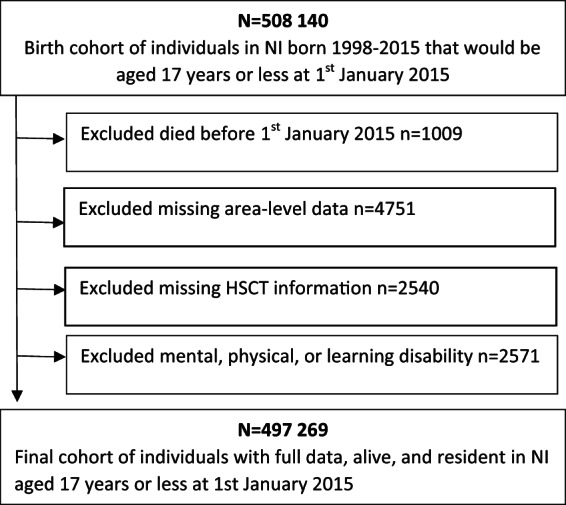
Flowchart of the cohort selection for the study of mental ill health in children in NI based on the level of contact with social services.

Figure 1 shows the cohort generation for the 497,269 children alive and resident in NI on January 1, 2015. A total of 2571 children recorded within SOSCARE as having a physical, mental or learning disability were excluded to allow a more robust analysis of the true association between demographic and social services related factors and mental ill health, without the possible confounding caused by existing disabilities or neurodevelopmental conditions. A further 7291 cases were removed because of missing area or Health and Social Trust indicators, leaving a total of 497,269 (98%) children.

#### Statistical analyses

Social care categories were compared by mental health outcomes and covariates using the chi-square statistic for categorical variables. Descriptive statistics were used to quantify the levels of contact children had with social services over their lifetime and the levels of contact in 2015. In order to keep the number of tables within limits, the main analyses in this paper only describe the mental health of children ‘currently’ (in 2015) in contact with social services according to the NIN, CIN and CIC groups (*n* = 23,975) compared to those never known (*n* = 411,477). The equivalent results including children with previous contact with social services but none in 2015 (*n* = 61,817) are available in Supplementary Tables S2–S4. Stepwise multilevel logistic regression models were constructed to examine the association between level of social care contact and each mental ill health indicator, adjusting for socio-demographic factors and clustering by Trust (HSCT). Sensitivity analyses were carried out identifying ≥3 prescriptions or items as the outcome for psychotropic medication uptake to rule out transient events. All analyses were conducted using STATA 15.0 software.

## Results

Of the study population, almost one child in every six (17.2%, *n* = 85,792) were currently or previously in contact with social services. Approximately one child in every 20 (4.8%, *n* = 23,975) were in contact with social services in 2015; 4876 (20.3%) NIN, 17,795 (74.2%) CIN and 1304 (5.4%) CIC. Table 1 shows their socio-demographic and social care characteristics (characteristics including those with previous social care contact are provided in Supplementary Table S2).

**Table 1.** Characteristics of children in NI aged 17 years or less in 2015 by level of contact with social services (*N* = 497,269^a^)

	Contact with social services in 2015								
	No contact		Not in need		Child in need		Child in care		
	*n* = 411,477 (82.7%)		*n* = 4876 (1.0%)		*n* = 17,795 (3.6%)		*n* = 1304 (0.3%)		*p*
Age									<0.001
0−10 years	269,061	65.4%	3009	61.7%	11,339	63.7%	489	37.5%	
11−17 years	142,416	34.6%	1867	38.3%	6456	36.3%	815	62.5%	
Sex									<0.001
Female	201,491	49.0%	2351	48.2%	8749	49.2%	668	51.2%	
Male	209,986	51.0%	2525	51.8%	9046	50.8%	636	48.8%	
Deprivation									
More deprived	164,510	40.0%	2674	54.8%	9953	55.9%	833	63.9%	<0.001
Less deprived	246,967	60.0%	2202	45.2%	7842	44.1%	471	36.1%	
Conurbation									<0.001
Rural	122,781	29.8%	819	16.8%	3498	19.7%	248	19.0%	
Intermediate	136,397	33.2%	1689	34.6%	7647	43.0%	556	42.6%	
Urban	152,299	37.0%	2368	48.6%	6650	37.4%	500	38.3%	
Reason referred									<0.001
Parent/guardian factors	–	–	–	–	14,352	80.6%	760	58.3%	
Well-being prejudiced	–	–	–	–	1454	8.2%	504	38.6%	
Other	–	–	–	–	1989	11.2%	40	3.1%	
Reason in care									–
Parent/guardian factors	–	–	–	–	–	–	321	24.6%	
Abuse/neglect	–	–	–	–	–	–	792	60.7%	
Other	–	–	–	–	–	–	191	14.7%	
Age first referral									<0.001
0−3 years	–	–	1761	36.1%	8310	46.7%	902	69.2%	
4−10 years	–	–	1754	36.0%	6259	35.2%	303	23.2%	
≥11 years	–	–	1361	27.9%	3226	18.1%	99	7.6%	
Age first in care									–
0−3 years	–	–	–	–	–	–	466	35.7%	
4−10 years	–	–	–	–	–	–	466	35.7%	
≥11 years	–	–	–	–	–	–	372	28.5%	
Number of referrals									<0.001
1	–	–	2664	54.6%	5303	29.8%	132	10.1%	
2−3	–	–	1233	25.3%	6491	36.5%	384	29.5%	
≥4	–	–	979	20.1%	6001	33.7%	788	60.4%	
Number care episodes									–
1	–	–	–	–	–	–	1037	79.5%	
2−3	–	–	–	–	–	–	223	17.1%	
≥4	–	–	–	–	–	–	44	3.4%	
Placement type									
Foster	–	–	–	–	–	–	766	58.7%	–
Kinship	–	–	–	–	–	–	175	13.4%	
Children’s home	–	–	–	–	–	–	53	4.1%	
Other^b^	–	–	–	–	–	–	310	23.8%	

a For brevity, children previously in contact with social services (*n* = 61,817 [12.4%]) not shown here but are included in Supplementary Table S2.

b Other includes at home with parent(s), specialist residential care, supported/temporary accommodation, juvenile justice/prison and unknown.

CIC in 2015 tended to be proportionately older than all other subgroups, but there was no substantial difference in gender distribution. The expected urban and socio-economic gradients in social services contact are also evident, with more children in contact with social services from deprived or urban areas compared to those never in contact with social services. Most CIN were referred to social services for parent/guardian factors, whilst over two-thirds (60.7%) of CIC in 2015 were in care due to abuse or neglect. A total of 69.2% of CIC were known to social services before the age of 4 years, with children deemed NIN referred at older ages. The majority of CIC had four or more referrals (60.4%), whilst the majority of children assessed as NIN had only one referral (54.6%). Only 4.1% of CIC in 2015 were most often in a children’s home, with children placed most frequently in foster (58.7%) or kinship care (13.4%).

### Relationship to mental ill health

Whilst children known to social services in 2015 account for only 4.8% of the cohort, they represent 18.6% of children who experienced mental ill health in 2015. Within the full birth cohort, children previously in contact with social services or in contact in 2015 accounted for 17.2% of the cohort, yet represented 49.5% of children who experienced mental ill health in 2015 (Supplementary Table S2). Overall, only 1.2% (*n* = 5970) of NI children had any mental ill health (as recorded by our measures) in 2015 (Supplementary Table S2).

Table 2 illustrates that mental ill health was lowest (0.7%) in children with no social care history, but rose from 4.4% and 4.0%, respectively, for NIN and CIN to 13.7% for CIC. Antidepressants were the most common psychotropic medication dispensed, and levels were 18 times higher for CIC (6.8%) compared to those with no social care history (0.4%), although part of this difference will be driven by the older age of the CIC cohort. Presentations with self-harm or ideation exhibited comparable gradients. The patterns for children with previous contact (Supplementary Table S2) are very similar, with the highest level of mental ill health in previous CIC and comparable levels between previous CIN and children previously assessed as NIN.

**Table 2.** Likelihood of mental ill health in children aged 17 years and under in NI according to the level of contact with social services in 2015

	Contact with social services in 2015			
	No contact	Not in need	Child in need	Child in care
Any mental ill health^a^				
% category	0.7%	4.4%	4.0%	13.7%
Unadjusted	Reference	6.05 (5.24−6.97)	5.76 (5.30−6.26)	21.89 (18.61−25.75)
Fully adjusted^b^	Reference	5.90 (5.10−6.83)	5.99 (5.50−6.53)	12.60 (10.63−14.95)
Antidepressants				
% usage	0.4%	1.9%	1.8%	6.8%
Unadjusted	Reference	5.16 (4.17−6.38)	5.02 (4.44−5.68)	20.65 (16.52−25.80)
Fully adjusted^b^	Reference	5.77 (4.62−7.20)	6.81 (5.98−7.76)	9.69 (7.63−12.30)
Anxiolytics				
% usage	0.2%	0.5%	0.5%	2.2%
Unadjusted	Reference	3.24 (2.17−4.84)	2.90 (2.32−3.64)	13.59 (9.27−19.92)
Fully adjusted^b^	Reference	3.04 (2.03−4.55)	2.82 (2.25−3.55)	9.02 (6.13−13.28)
Hypnotics				
% usage	0.2%	1.1%	1.1%	3.3%
Unadjusted	Reference	4.77 (3.60−6.32)	5.52 (4.73−6.44)	15.94 (11.67−21.78)
Fully adjusted^b^	Reference	4.35 (3.27−5.77)	5.14 (4.40−6.01)	10.68 (7.79−14.66)
Antipsychotics				
% usage	0.1%	0.5%	0.6%	3.5%
Unadjusted	Reference	7.38 (4.89−11.14)	8.44 (6.69−10.65)	55.13 (39.98−76.02)
Fully adjusted^b^	Reference	6.90 (4.55−10.45)	8.94 (7.05−11.33)	27.67 (19.88−38.52)
Any psych meds				
% usage	0.7%	3.2%	3.1%	10.4%
Unadjusted	Reference	4.53 (3.84−5.34)	4.54 (4.14−4.98)	16.72 (13.93−20.07)
Fully adjusted^b^	Reference	4.35 (3.67−5.15)	4.66 (4.24−5.13)	9.54 (7.89−11.53)
Self-harm/ideation				
% category	0.1%	1.9%	1.7%	6.5%
Unadjusted	Reference	29.22 (22.94−37.22)	27.77 (23.46−32.87)	113.4 (88.12−146.01)
Fully adjusted^b^	Reference	29.51 (23.01−37.85)	31.24 (26.23−37.21)	52.30 (40.10−68.22)

Data represent odds ratios (and 95% confidence intervals) from multilevel logistic regression models adjusting for clustering by Health and Social Care Trust

a Any psychotropic medication, self-harm or ideation or psychiatric hospital admission.

b Fully adjusted MLM for child age and sex, area-level income deprivation and conurbation and clustering by HSCT

The multilevel logistic regression estimates confirm the descriptive patterns of increased mental ill health with level of social services contact (Table 2). In the fully adjusted models, CIC were over 12 times more likely to experience any mental ill health outcome (odds ratio [OR] 12.60 [95% confidence interval (CI) 10.63–14.95]) than their peers who had no contact. CIC were over 9 times more likely to receive antidepressants (OR 9.69 [95% CI 7.63–12.30]), 27 times more likely to receive antipsychotics (OR 27.67 [95% CI 19.88–38.52]) and 52 times more likely to present with self-harm/ideation (OR 52.30 [95% CI 40.10–68.22]) compared to children with no contact. Children assessed as NIN or CIN in 2015 had similar magnitude of risk across all mental ill health indicators and were over five times more likely to experience any mental ill health compared with peers with no contact (OR 5.90 [95% CI 5.10–6.83] and OR 5.99 [95% CI 5.50–6.53], respectively). Children previously known to social services but with no contact in 2015 were still more likely to have poor mental health compared with those with no social care history (Supplementary Table S3). Children previously CIC but no longer in care in 2015 were still over five times more likely (OR 5.58 [95% CI 4.68–6.67]) to have any mental ill health outcome compared to those never in contact with social services. Sensitivity analyses were carried out using ≥3 prescriptions/items for each type of psychotropic medication in 2015 as the outcome to rule out transient events and ‘one-off’ prescriptions. These analyses yielded similar trends and effect sizes as observed in the main analyses (Supplementary Table S4).

### Social care experiences and mental ill health in childhood

Separate multilevel logistic regression models examined which social care experiences were associated with mental ill health within CIN (Table 3) and CIC (Table 4) in 2015 only (*n* = 17,795 and *n* = 1304, respectively). Outcomes were restricted to receipt of any psychotropic medication and any mental ill health outcome due to small numbers for data disclosure. After full adjustment for socio-demographic and social care characteristics, only the variables ‘reason referred’ and ‘number of referrals’ remained positively associated with mental ill health in CIN (Table 3). CIN referred due to parent or guardian factors (primarily carer requires support) were more likely to receive psychotropic medication (OR 1.72 [95% CI 1.11–2.67]) or experience any mental ill health outcome (OR 1.86 [95% CI 1.25–2.77]) compared with CIN referred most frequently for ‘other’ reasons. CIN referred on four or more occasions were more likely to receive psychotropic medication (OR 1.58 [95% CI 1.17–2.12]) or experience any mental ill health outcome (OR 1.66 [95% CI 1.27–2.16]), compared to CIN with a single referral episode.

**Table 3.** Attributes of social care and likelihood of mental ill health among the 17,795 children in need in NI in 2015

	Any psychotropic medication		Any mental ill health outcome^a^	
Social care characteristics	Model 1 (unadjusted)	Model 2 (adjusted)	Model 1 (unadjusted)	Model 2 (adjusted)
Reason referred				
Other	Reference	Reference	Reference	Reference
Parent/guardian factors	2.33 (1.52−3.58)	1.72 (1.11−2.67)	2.51 (1.71−3.69)	1.86 (1.25−2.77)
Well-being prejudiced	1.99 (1.19−3.34)	1.36 (0.79−2.32)	1.98 (1.24−3.15)	1.38 (0.85−2.25)
Age first referred				
0−3 years	Reference	Reference	Reference	Reference
4−10 years	2.31 (1.84−2.90)	1.00 (0.78−1.29)	2.38 (1.94−2.93)	1.02 (0.81−1.27)
≥11 years	5.26 (4.20−6.60)	1.04 (0.76−1.43)	6.50 (5.32−7.95)	1.23 (0.93−1.63)
Number of referrals				
1	Reference	Reference	Reference	Reference
2−3	1.41 (1.10−1.81)	1.13 (0.87−1.48)	1.56 (1.25−1.93)	1.31 (1.03−1.65)
≥4	2.50 (1.98−3.16)	1.58 (1.17−2.12)	2.39 (1.94−2.93)	1.66 (1.27−2.16)
*χ*^2^ (MLM vs. logistic)		7.63		6.65
*P*		0.0029		0.0050
ICC		0.0118		0.00859

Data represent odds ratios and (95% confidence intervals) from multilevel logistic regression models adjusting for clustering by Health and Social Care Trust; Health and Social Care Trust *n* = 5.

a Any psychotropic medication, self-harm or ideation or psychiatric hospital admission; Model 1 unadjusted; Model 2 adjusted for all social care variables plus child age, sex, area-level deprivation and conurbation; ICC, intraclass correlation.

The social care history characteristics associated with mental ill health in CIC are shown in Table 4. In the fully adjusted models, only number of care episodes and most frequent care placement type remained positively associated with likelihood of mental ill health. CIC that experienced four or more care episodes were 2.8 times more likely to receive psychotropic medication compared to CIC with a single episode (OR 2.81 [95% CI 1.20–6.60]). Children placed most frequently in a children’s home were over twice as likely to receive psychotropic medication and experience any mental ill health outcome, compared to those placed most frequently in non-kinship foster care (OR 2.42 [95% CI 1.16–5.05] and OR 2.64 [95% CI 1.33–5.24], respectively).

**Table 4.** Attributes of social care and likelihood of mental ill health among 1304 children in care in NI in 2015

	Any psychotropic medication		Any mental ill health outcome^a^	
Social care characteristics	Model 1 (unadjusted)	Model 2 (adjusted)	Model 1 (unadjusted)	Model 2 (adjusted)
Reason in care				
Other	Reference	Reference	Reference	Reference
Parent/guardian factors	1.47 (0.87−2.49)	1.08 (0.51−2.29)	1.78 (1.11−2.86)	1.65 (0.81−3.36)
Abuse/neglect	0.57 (0.34−0.96)	0.68 (0.32−1.41)	0.57 (0.36−0.91)	0.84 (0.42−1.70)
Age first in care				
0−3 years	Reference	Reference	Reference	Reference
4−10 years	1.22 (0.72−2.07)	0.75 (0.42−1.35)	1.19 (0.75−1.89)	0.74 (0.44−1.25)
≥11 years	4.10 (2.57−6.53)	1.20 (0.66−2.17)	4.34 (2.87−6.55)	1.28 (0.75−2.18)
**Number of care episodes**				
1	Reference	Reference	Reference	Reference
2−3	1.68 (1.09−2.59)	1.27 (0.78−2.07)	1.91 (1.31−2.79)	1.43 (0.92−2.22)
≥4	3.14 (1.49−6.60)	2.81 (1.20−6.60)	2.68 (1.31−5.49)	2.12 (0.94−4.81)
Placement type				
Foster	Reference	Reference	Reference	Reference
Kinship	0.89 (0.50−1.60)	1.31 (0.69−2.49)	0.67 (0.39−1.16)	0.94 (0.51−1.71)
Children’s home	4.84 (2.60−9.03)	2.42 (1.16−5.05)	5.96 (3.31−10.75)	2.64 (1.33−5.24)
Other	0.98 (0.61−1.53)	1.44 (0.84−2.46)	0.81 (0.53−1.22)	1.13 (0.69−1.85)
*χ*^2^ (MLM vs. logistic)		1.42		0.19
*P*		0.1168		0.3327
*ICC*		0.0138		0.00427

Data represent odds ratios and (95% confidence intervals) from multilevel logistic regression models adjusting for clustering by Health and Social Care Trust

Health and Social Care Trust *n* = 5;

a Any psychotropic medication, self-harm or ideation or psychiatric hospital admission; Model 1 unadjusted; Model 2 adjusted for all social care variables plus child age, sex, area-level deprivation and conurbation; ICC, intraclass correlation

## Discussion

This is the first UK population-wide study to examine the extent of all contact with children’s social services and the associated mental health status of the children concerned. In 2015, almost 1 in 20 (4.8%) of the children in NI aged 17 years and under were in contact with social services that year. If children with previous contact are also included, this proportion increases to more than one in six (17.2%, *n* = 85,792). It is difficult to find comparable population-level data, as existing data use different methods, estimates and timeframes (Department for Education, 2019; Fitzsimons *et al.*, 2022).

### Contact with social services and mental ill health

The study confirms the association between social services contact and mental ill health in children, though it extends the examination beyond the more often studied CIC to include all levels of contact with social services. It clearly demonstrates that the differences between those with social care contact and those without are both large and increase according to the level and frequency of social services contact and intervention and that the relationship is robust across a range of mental ill health indicators. These support previous research which found high rates of antidepressant prescribing, hospital presenting self-harm and mental health diagnoses in CIC compared to unexposed peers (Allik *et al.*, 2022; Bronsard *et al.*, 2016; Fleming *et al.*, 2021; Green *et al.*, 2020; Maclean *et al.*, 2019).

A graded relationship between tier of contact with social services and mental ill health likely reflects the degree of exposure to early life adversity, as the majority of children entering care do so because of abuse or neglect, and the removal of a child is a measure of last resort in cases of serious threat to safety or well-being. In addition to pre-care adversity, disrupted attachments and in care experiences such as placement instability are associated with poor mental health (Rutter, 2000). Higher rates of psychotropic prescribing and hospital presenting self-harm among children in care may be driven in part by differential access to services, for example through closer supervision by social care workers. However, there is also considerable evidence of barriers to help-seeking and inadequate mental health support for children in care (Powell *et al.*, 2021). Mandatory screening for mental health problems in children in care is absent in NI and most jurisdictions (Tarren-Sweeney *et al.*, 2019), and where it does exist (e.g. in England), the results rarely inform the development of a child’s care plan (Care Quality Commission, 2016). Recent policy in NI commits to introducing a more holistic approach to health assessment at the point of entry to care, with an increased focus on mental health needs (Department of Health, 2021).

Although the risk of mental ill health is highest in CIC, who have a 52-fold increased likelihood of self-harm/ideation, this represents a small exposure group and four times as many children within the NIN and CIN groups presented to ED with self-harm/ideation. We also show that children assessed as NIN who received no social care have generally equivalent levels of mental ill health to those defined as CIN and subsequently received support or protection measures at home. This highlights for the first time an important ‘at-risk’ population, the NIN, who may benefit from targeted interventions. The only potentially comparable prior research examines children subject to a child protection investigation where the risk threshold was not met, or the maltreatment was unsubstantiated, finding that they too are more likely to experience mental ill health than children with no child protection contact (Green *et al.*, 2020; Maclean *et al.*, 2019; O’Hare *et al.*, 2021).

### Social care experiences and mental ill health

The prominent risk marker for mental ill health in CIN and CIC is a higher number of referrals/episodes. Multiple CIN referrals in childhood may suggest that child or family support needs were not fully addressed or that interventions were inadequate or too short-term to be effective (Devaney, 2009; Hood *et al.*, 2020; MacAlister, 2021). Extended exposure to childhood adversity, and exposure to multiple types of adversity, are particularly damaging to mental health (Hughes *et al.*, 2017; Steptoe *et al.*, 2019). A higher number of care episodes (four or more) is associated with a twofold increase in likelihood of psychotropic medication uptake for CIC. This may be indicative of unsuccessful reunifications or of unstable placements, which can both exacerbate child mental ill health and be the result of child mental health or behavioural problems (Fawley-King and Snowdon 2012; Harkin and Houston, 2016; Jones *et al.*, 2011).

The finding that children in residential care are at greater risk of mental ill health than children in foster care is consistent with previous research (Li *et al.*, 2019). However, it is unclear if this reflects baseline child characteristics, as residential care is used most often for older children with higher needs (Chor, 2013; Li *et al.*, 2019; Thoburn, 2009). There is a role for this type of placement, though the quality of residential care and mental health support is key (Thoburn and Courtney, 2011). While previous studies have found that the risk of mental ill health was higher for children who had experienced maltreatment (Negriff, 2020), this study found that the reason for entering care did not stratify risk amongst children in care.

### Strengths and limitations

This is the first population-wide study of childhood contact with social services, which includes all levels of contact with social services and even identifies those assessed as NIN. It utilizes several indicators of mental ill health, reducing the heterogeneity in self-report measures and screening tools that can produce widely divergent estimates of mental ill health. It identifies for the first time the magnitude of population contact with children’s social services, quantifying that in any 1 year approximately one in 20 children are in contact with social services with one in six having previous or current contact with social services. These children in contact or previously in contact with social services in 2015 represent 49.5% of children who experienced mental ill health that year.

Identifying at-risk groups is of vital importance in the targeting of resources or interventions. However, there are also several limitations of note. The most significant limitation is an inability to move beyond association. The cross-sectional design obviates the exploration of important counterfactuals, i.e. whether mental health would have been better or worse in the absence of social services intervention or that the gradients of mental ill health and level of social service contact are not due to confounding by indication. However, in the absence of experimental evidence, it may be possible that examination of differences or changes in policy/practice approaches between countries or administrative units within countries may be helpful. The indicators of mental ill health utilized are imperfect. The prescribing data does not contain information on the conditions or symptoms a drug is prescribed for, and these medications are sometimes used ‘off-label’ in young children for neurodevelopmental disorders or treatment of tics (Sharma *et al.*, 2016). Establishing a clear link between categories of psychotropic medication usage and specific mental disorders is often unfeasible, though studies have shown it is highly likely that their usage indicates mental health problems (Cleland *et al.*, 2018; Gardarsdottir *et al.*, 2009). The hospital data do not contain diagnostic codes and could include cases unrelated to mental health problems, such as respite admission for children with intellectual disabilities. However, we mitigate against this by excluding individuals with a recorded mental or learning disability from the cohort. It should also be recognized that identifying mental ill health in children is difficult, and the measures used in the present study are relatively rare in young children. Consequently, it is likely that this study is an underestimation of the mental health burden, though this limitation also applies to the whole population.

### Implications

The study demonstrates that children known to social services account for a large and disproportionate amount of the poor mental health in the child population. This is important as it is recognized that most mental ill health starts in early years and carries through into adulthood (Pine and Fox, 2015). The mental ill health of these children represents a huge current and future burden for society. These results have numerous implications for children’s social care and broader policy and highlight a potential missed opportunity for intervention within this population of children. The major research focus to date has been on children in care, but the larger absolute numbers of individuals with mental ill health known to social services are within the CIN and NIN groups. Further research will also be needed to determine if there are critical periods when children are particularly at risk of poor mental health. Tackling the disproportionate burden of mental ill health among children in contact with social services, including those deemed NIN that do not meet the threshold for services, requires cross-departmental and multi-agency social programs that promote and protect the well-being of children, families and communities. The especially high burden of mental ill health among care experienced children suggests the need for routine screening within this group, with results used to inform care plans, and aligned with access to relevant services and evidence-informed therapeutic interventions.

## Data Availability

The data underlying this article were provided by the Honest Broker Service in the Trusted Research Environment for Health and Social Care (HSC), Northern Ireland, which provides approved researchers with access to linked, de-identified health and social care data. Data are available for research projects in the public interest that relate to Health and Social Care, subject to approval by the Honest Broker Service Governance Board (for information contact honestbrokerservice@hscni.net). Additional approvals are required to access data collected by the Northern Ireland Registry of Self-harm. Access to Registry data is available via the Honest Broker Service once approval from all Health and Social Care Trusts in Northern Ireland has been received. To access data from the Northern Ireland Registry of Self-harm, please contact the Public Health Agency (reception.pha@hscni.net).

## Supplementary material

For supplementary material accompanying this paper visit http://doi.org/10.1017/S2045796023000276. click here to view supplementary material
