# Supplementary material for: The mental health of all children in contact with social services: a population-wide record-linkage study in Northern Ireland
Source: Epidemiol Psychiatr Sci. 2023 May 16;32:e35. doi: 10.1017/S2045796023000276 (PMC10227534; doi:10.1017/S2045796023000276)
Supplement: Supplementary file 1 [file epssup.zip › S2045796023000276sup001.docx]

**Supplementary Table S1** Full list of datasets, variables and coding used to determine the period prevalence of mental ill-health of children aged 17 years or less in Northern Ireland in 2015 based on level of contact with social services

| **Variable** | **Description** | **Dataset** |
| --- | --- | --- |
| ***Individual characteristics*** |  |  |
| Child sex | Male / female | NHAIS |
| Child age | Generated from date of birth and assigned at 1^st^ January 2015. Categorised into ages 0-10 years and 11-17 years for descriptive purposes, to distinguish between children (pre-and-during primary school) and post-primary school aged children in the cohort. Utilised as a continuous variable in regression analyses due to the relative rarity of the mental health indicators in very young children. | NHAIS |
| ***Area-level characteristics*** |  |  |
| Conurbation | Indicator of level of conurbation assigned to address in 2010 or at birth if born later. Data custodians generate settlement band within NHAIS using postcode information from patient address, linked to the Northern Ireland Research and Statistics Agency (NISRA) classification of settlements in Northern Ireland. Settlement bands range from band A-H. Level of conurbation was categorised into three groups: urban (bands A-B, cities Belfast and Derry); intermediate (bands C-F, towns and intermediate settlements); and rural (bands G-H, villages and settlements <1,000 people and open countryside). | NHAIS |
| Area-level deprivation | Indicator of area-level income deprivation assigned to address in 2010, or address at birth if born later. Area-level income deprivation generated by data custodians using patient address from NHAIS and the Income domain of the 2010 Northern Ireland Multiple Deprivation Measure (NIMDM). Areas were ranked from the most to least deprived, and divided into quintiles. Quintiles were subsequently categorised into two groups, less deprived and more deprived. | NHAIS |
| Health and Social Care Trust (HSCT) | HSCT identifier (n=5) assigned to address in 2010. | NHAIS |
| ***Social care characteristics*** |  |  |
| Exposure history | Level of interaction with social services in childhood coded into four mutually exclusive categories based on highest level (no involvement; not in need (NIN); child in need (CIN); child in care (CIC)). NIN was defined as children referred to social services but assessed not in need of help or protection, in every interaction. CIN was defined as children services in receipt of social care services i.e. on a Child in Need Plan and/or subject to child protection investigation/registration. CIC was defined as Looked After Children in care, including for respite purposes. It was necessary to examine incident cases of CIN in 2015 (i.e. new referrals), but prevalent cases of CIC in 2015, because the SOSCARE data contained no ‘case closed’ date for CIN. NIN and CIN were identified by a referral episode with social services at any point during 2015. CIC in 2015 were identified if they were in care at any point during 2015. | SOSCARE |

**Supplementary Table S1** *continued*

| Reason involved with social services | Most common reason for involvement with social services from all possible reasons coded in SOSCARE was established for each child across their full social care history and subsequently coded into three categories (parent or guardian factors; wellbeing prejudiced; other). Children referred but assessed Not in Need in every interaction do not have a reason.  Parent or guardian factors includes: no one exercising parental responsibility; carer temporarily unable to care; carer requires support; family bereavement.  Wellbeing prejudiced includes: subject to a child protection investigation; wellbeing significantly prejudiced; welfare prejudiced homelessness; with caring duties impairing development; request for financial assistance.  Other includes: offending a significant feature; ceasing to be 'looked after' by Trust; awaiting assessment; other/unknown | SOSCARE |
| --- | --- | --- |
| Reason in care | Most common reason for placement in care coded in SOSCARE was established for each child across their full care history and subsequently coded into three categories (parent or guardian factors; abuse or neglect; other).  Parent or guardian factors incudes: confinement of parent/carer; death of parent/carer; imprisonment of parent/carer; physical illness parent/carer; mental illness parent/carer; predicted parental inadequacy; desertion by parent/carer; beyond control of parent/carer.  Abuse or neglect includes: neglect; suspected abuse; emotional abuse; physical abuse; sexual abuse.  Other includes: missing; access; pre-adoption; family relief/respite; relief of parental stress; criminal offence child; power of courts; self-referral by child; schedule 1 offender household member. | SOSCARE |
| Child age at first referral | Child age at their first referral to social services was calculated by using date of birth and first recorded SOSCARE episode and subsequently coded into three categories (0-3 years; 4-10 years; ≥11 years). | SOSCARE |
| Child age at first entry to care | Age at first placement in care was calculated by using date of birth and first recorded SOSCARE episode of care and subsequently coded into three categories (0-3 years; 4-10 years; ≥11 years) | SOSCARE |
| Number of referral episodes | The number of unique episodes recorded in SOSCARE was used to provide an estimate of the number of times a child was referred to social services, and subsequently coded into three categories (1; 2-3; ≥4) | SOSCARE |
| Number of care episodes | Month and year of each care episode was used to calculate the number of unique episodes, and subsequently coded into three categories (1; 2-3; ≥4). New episodes may relate to new referrals after a case closure, but also a change in the legal status of the child or a change in care provision such as a placement move. | SOSCARE |
| Placement type | Most frequently recorded care placement type (kinship foster; non-kinship foster; children’s home; other). Other includes supported/temporary accommodation; juvenile justice/prison; at home with parent(s). | SOSCARE |

**Supplementary Table S1** *continued*

| ***Physical disability*** | Yes/No indicator of the individual having a record in the SOSCARE “Physical Disability” module to identify potential disability. Used to delineate cohort. | SOSCARE |
| --- | --- | --- |
| ***Mental disability*** | Yes/No indicator of the individual having a record in the SOSCARE “Mental Disability” module to identify potential disability. Used to delineate cohort. | SOSCARE |
| ***Learning disability*** | Yes/No indicator of the individual having a record in the SOSCARE “Learning Disability” module to identify potential disability. Used to delineate cohort. | SOSCARE |
| ***Mental ill-health indicator*** |  |  |
| Antidepressants | Yes/No indicator for ever/never prescribed at least one prescription or item in 2015. British National Formulary 69 (BNF) classifications 4.3.1; 4.3.2; 4.3.3; & 4.3.4 | EPD |
| Anxiolytics | Yes/No indicator for ever/never prescribed at least one prescription or item in 2015. BNF 4.1.2 | EPD |
| Antipsychotics | Yes/No indicator for ever/never prescribed at least one prescription or item at least one prescription or item in 2015. BNF 4.2.1 & 4.2.2 | EPD |
| Hypnotics | Yes/No indicator for ever/never prescribed at least one prescription or item in 2015. BNF 4.1.1 | EPD |
| Any psychotropic medication | Yes/No indicator for ever/never prescribed any of the four categories of psychotropic medication in 2015 | EPD |
| Self-harm or self-harm/suicidal ideation | Yes/No indicator for ever/never presented to an Emergency Department in 2015 following self-harm or self-harm/suicidal ideation | NIRSH |
| Psychiatric hospital admission | Yes/No indicator for ever/never experienced a psychiatric hospital admission to a general ward or specialist unit in 2015. | Hospital data |
| Any mental ill-health | Yes/No indicator for any mental ill-health in 2015 (any of the four categories of psychotropic medication / self-harm or ideation / psychiatric hospital admission) | EPD, NIRSH, Hospital data |

*NHAIS, National Health Applications and Infrastructure Services; SOSCARE, Social Services Client Administration and Retrieval Environment; EPD, Enhanced Prescribing Database; NIRSH, Northern Ireland Registry of Self-Harm*
