# Supplementary material for: The mental health of all children in contact with social services: a population-wide record-linkage study in Northern Ireland
Source: Epidemiol Psychiatr Sci. 2023 May 16;32:e35. doi: 10.1017/S2045796023000276 (PMC10227534; doi:10.1017/S2045796023000276)
Supplement: Supplementary file 1 [file epssup.zip › S2045796023000276sup002.docx]

**Supplementary Table S2** Characteristics of children in Northern Ireland aged 17 years or less in 2015 and prevalence of mental ill-health by level of contact with social services (N=497 269)

|  | **Total cohort** | | **No contact** | | **NIN before 2015** | | **CIN before 2015** | | **CIC before 2015** | | **NIN in 2015** | | **CIN in 2015** | | **CIC in 2015** | | ***p*** |
| --- | --- | --- | --- | --- | --- | --- | --- | --- | --- | --- | --- | --- | --- | --- | --- | --- | --- |
| **Characteristic** | N=497 269 (100%) | | n=411 477 (82.7%) | | n=12 478 (2.5%) | | n=47 164 (9.5%) | | n=2175 (0.4%) | | n=4876 (1.0%) | | n=17 795 (3.6%) | | n=1304 (0.3%) | |  |
| **Age at 1^st^ Jan 2015** |  |  |  |  |  |  |  |  |  |  |  |  |  |  |  |  | <0.001 |
| *0-10 years* | 314 263 | (63.2%) | 269 061 | (65.4%) | 5761 | (46.2%) | 23 728 | (50.3%) | 876 | (40.3%) | 3009 | (61.7%) | 11 339 | (63.7%) | 489 | (37.5%) |  |
| *11-17 years* | 183 006 | (36.8%) | 142 416 | (34.6%) | 6717 | (53.8%) | 23 436 | (49.7%) | 1299 | (59.7%) | 1867 | (38.3%) | 6456 | (36.3%) | 815 | (62.5%) |  |
| **Sex** |  |  |  |  |  |  |  |  |  |  |  |  |  |  |  |  | <0.001 |
| *Female* | 242 951 | (48.9%) | 201 491 | (49.0%) | 5812 | (46.6%) | 22 837 | (48.4%) | 1043 | (48.0%) | 2351 | (48.2%) | 8749 | (49.2%) | 668 | (51.2%) |  |
| *Male* | 254,318 | (51.1%) | 209 986 | (51.0%) | 6666 | (53.4%) | 24 327 | (51.6%) | 1132 | (52.0%) | 2525 | (51.8%) | 9046 | (50.8%) | 636 | (48.8%) |  |
| **Income deprivation** |  |  |  |  |  |  |  |  |  |  |  |  |  |  |  |  | <0.001 |
| *More deprived* | 210 872 | (42.4%) | 164 510 | (40.0%) | 6343 | (50.8%) | 25 291 | (53.6%) | 1268 | (58.3%) | 2674 | (54.8%) | 9953 | (55.9%) | 833 | (63.9%) |  |
| *Less deprived* | 286 397 | (57.6%) | 246 967 | (60.0%) | 6135 | (49.2%) | 21 873 | (46.4%) | 907 | (41.7%) | 2202 | (45.2%) | 7842 | (44.1%) | 471 | (36.1%) |  |
| **Conurbation** |  |  |  |  |  |  |  |  |  |  |  |  |  |  |  |  | <0.001 |
| *Rural* | 140 707 | (28.3%) | 122 781 | (29.8%) | 2514 | (20.2%) | 10 534 | (22.3%) | 313 | (14.4%) | 819 | (16.8%) | 3498 | (19.7%) | 248 | (19.0%) |  |
| *Intermediate* | 171 349 | (34.5%) | 136 397 | (33.2%) | 4918 | (39.4%) | 19 100 | (40.5%) | 1042 | (47.9%) | 1689 | (34.6%) | 7647 | (43.0%) | 556 | (42.6%) |  |
| *Urban* | 185 213 | (37.2%) | 152 299 | (37.0%) | 5046 | (40.4%) | 17 530 | (37.2%) | 820 | (37.7%) | 2368 | (48.6%) | 6650 | (37.4%) | 500 | (38.3%) |  |
| **Reason referred** |  |  |  |  |  |  |  |  |  |  |  |  |  |  |  |  | <0.001 |
| *Parent/guardian factors* | -- | -- | -- | -- | -- | -- | 37 934 | (80.4%) | 1361 | (62.5%) | -- | -- | 14 352 | (80.6%) | 760 | (58.3%) |  |
| *Wellbeing prejudiced* | -- | -- | -- | -- | -- | -- | 4998 | (10.6%) | 667 | (30.7%) | -- | -- | 1454 | (8.2%) | 504 | (38.6%) |  |
| *Other* | -- | -- | -- | -- | -- | -- | 4232 | (9.0%) | 147 | (6.8%) | -- | -- | 1989 | (11.2%) | 40 | (3.1%) |  |

**Supplementary S2** continued

|  | **Total cohort** | | **No contact** | | **NIN before 2015** | | **CIN before 2015** | | **CIC before 2015** | | **NIN in 2015** | | **CIN in 2015** | | **CIC in 2015** | | ***p*** |
| --- | --- | --- | --- | --- | --- | --- | --- | --- | --- | --- | --- | --- | --- | --- | --- | --- | --- |
| **Characteristic** | N=497 269 (100%) | | n=411 477 (82.7%) | | n=12 478 (2.5%) | | n=47 164 (9.5%) | | n=2175 (0.4%) | | n=4876 (1.0%) | | n=17 795 (3.6%) | | n=1304 (0.3%) | |  |
| **Reason in care** |  |  |  |  |  |  |  |  |  |  |  |  |  |  |  |  | -- |
| *Parent/guardian factors* | -- | -- | -- | -- | -- | -- | -- | -- | 501 | (23.0%) | -- | -- | -- | -- | 321 | (24.6%) |  |
| *Abuse/neglect* | -- | -- | -- | -- | -- | -- | -- | -- | 1222 | (56.2%) | -- | -- | -- | -- | 792 | (60.7%) |  |
| *Other* | -- | -- | -- | -- | -- | -- | -- | -- | 452 | (20.8%) | -- | -- | -- | -- | 191 | (14.7%) |  |
| **Age at first referral** |  |  |  |  |  |  |  |  |  |  |  |  |  |  |  |  | <0.001 |
| *0-3 years* | -- | -- | -- | -- | 4688 | (37.6%) | 20 815 | (44.1%) | 1605 | (73.8%) | 1761 | (36.1%) | 8310 | (46.7%) | 902 | (69.2%) |  |
| *4-10 years* | -- | -- | -- | -- | 5262 | (42.2%) | 19 330 | (41.0%) | 461 | (21.2%) | 1754 | (36.0%) | 6259 | (35.2%) | 303 | (23.2%) |  |
| *≥11 years* | -- | -- | -- | -- | 2528 | (20.3%) | 7019 | (14.9%) | 109 | (5.0%) | 1361 | (27.9%) | 3226 | (18.1%) | 99 | (7.6%) |  |
| **Age first in care** |  |  |  |  |  |  |  |  |  |  |  |  |  |  |  |  | -- |
| *0-3 years* | -- | -- | -- | -- | -- | -- | -- | -- | 1208 | (55.5%) | -- | -- | -- | -- | 466 | (35.7%) |  |
| *4-10 years* | -- | -- | -- | -- | -- | -- | -- | -- | 667 | (30.7%) | -- | -- | -- | -- | 466 | (35.7%) |  |
| *≥11 years* | -- | -- | -- | -- | -- | -- | -- | -- | 300 | (13.8%) | -- | -- | -- | -- | 372 | (28.5%) |  |
| **Number of referrals** |  |  |  |  |  |  |  |  |  |  |  |  |  |  |  |  | <0.001 |
| *1* | -- | -- | -- | -- | 8964 | (71.8%) | 23 291 | (49.4%) | 56 | (2.6%) | 2664 | (54.6%) | 5303 | (29.8%) | 132 | (10.1%) |  |
| *2-3* | -- | -- | -- | -- | 2430 | (19.5%) | 16 269 | (34.5%) | 515 | (23.7%) | 1233 | (25.3%) | 6491 | (36.5%) | 384 | (29.5%) |  |
| *≥4* | -- | -- | -- | -- | 1084 | (8.7%) | 7604 | (16.1%) | 1604 | (73.8%) | 979 | (20.1%) | 6001 | (33.7%) | 788 | (60.4%) |  |
| **Number care episodes** |  |  |  |  |  |  |  |  |  |  |  |  |  |  |  |  | -- |
| *1* | -- | -- | -- | -- | -- | -- | -- | -- | 1741 | (80.0%) | -- | -- | -- | -- | 1037 | (79.5%) |  |
| *2-3* | -- | -- | -- | -- | -- | -- | -- | -- | 339 | (15.6%) | -- | -- | -- | -- | 223 | (17.1%) |  |
| *≥4* | -- | -- | -- | -- | -- | -- | -- | -- | 95 | (4.4%) | -- | -- | -- | -- | 44 | (3.4%) |  |
| **Placement type** |  |  |  |  |  |  |  |  |  |  |  |  |  |  |  |  | -- |
| *Foster* | -- | -- | -- | -- | -- | -- | -- | -- | 1213 | (55.8%) | -- | -- | -- | -- | 766 | (58.7%) |  |
| *Kinship* | -- | -- | -- | -- | -- | -- | -- | -- | 178 | (8.2%) | -- | -- | -- | -- | 175 | (13.4%) |  |
| *Children’s home* | -- | -- | -- | -- | -- | -- | -- | -- | 38 | (1.8%) | -- | -- | -- | -- | 53 | (4.1%) |  |
| *Other ^a^* | -- | -- | -- | -- | -- | -- | -- | -- | 746 | (34.3%) | -- | -- | -- | -- | 310 | (23.8%) |  |

**Supplementary Table S2** continued

|  | **Total cohort** | | **No contact** | | **NIN before 2015** | | **CIN before 2015** | | **CIC before 2015** | | **NIN in 2015** | | **CIN in 2015** | | **CIC in 2015** | | ***p*** |
| --- | --- | --- | --- | --- | --- | --- | --- | --- | --- | --- | --- | --- | --- | --- | --- | --- | --- |
| **Characteristic** | N=497 269 (100%) | | n=411 477 (82.7%) | | n=12 478 (2.5%) | | n=47 164 (9.5%) | | n=2175 (0.4%) | | n=4876 (1.0%) | | n=17 795 (3.6%) | | n=1304 (0.3%) | |  |
| *Any mental ill-health ^b^* | 5970 | (1.20%) | 3016 | (0.73%) | 334 | (2.68%) | 1365 | (2.89%) | 144 | (6.62%) | 214 | (4.39%) | 718 | (4.03%) | 179 | (13.73%) | <0.001 |
| *Antidepressants* | 2988 | (0.60%) | 1472 | (0.36%) | 196 | (1.57%) | 755 | (1.60%) | 70 | (3.22%) | 93 | (1.91%) | 314 | (1.76%) | 88 | (6.75%) | <0.001 |
| *Anxiolytics* | 1044 | (0.21%) | 667 | (0.16%) | 44 | (0.35%) | 183 | (0.39%) | 12 | (0.55%) | 25 | (0.51%) | 85 | (0.48%) | 28 | (2.15%) | <0.001 |
| *Antipsychotics* | 660 | (0.13%) | 272 | (0.07%) | 35 | (0.28%) | 157 | (0.33%) | 29 | (1.33%) | 25 | (0.51%) | 97 | (0.55%) | 45 | (3.45%) | <0.001 |
| *Hypnotics* | 1786 | (0.36%) | 886 | (0.22%) | 103 | (0.83%) | 456 | (0.97%) | 44 | (2.02%) | 52 | (1.07%) | 202 | (1.14%) | 43 | (3.30%) | <0.001 |
| *Any psych meds* | 5423 | (1.09%) | 2866 | (0.70%) | 315 | (2.52%) | 1290 | (2.74%) | 119 | (5.47%) | 154 | (3.16%) | 544 | (3.06%) | 135 | (10.35%) | <0.001 |
| *Self-harm/ideation* | 959 | (0.19%) | 255 | (0.06%) | 30 | (0.24%) | 160 | (0.34%) | 42 | (1.93%) | 92 | (1.89%) | 295 | (1.66%) | 85 | (6.52%) | <0.001 |

*Percentages may not exactly equal 100.0% due to rounding; NIN not in need; CIN child in need; CIC child in care; ^a^ Other includes at home, specialist residential care, supported/temporary accommodation, juvenile justice/prison and unknown; ^b^Any psychotropic medication, self-harm or ideation, or psychiatric hospital admission; Children previously known to social services or known in 2015 accounted for 17.2% of the cohort, yet represented 49.5% of children that experienced mental ill-health (percentages calculated from data in Table S2: n=2,954 cases of any mental ill-health out of n=5,970 total cases were in children known to social services, 2,954/5,970*100=49.5%).*
